# Formulation Development for Transdermal Delivery of Raloxifene, a Chemoprophylactic Agent against Breast Cancer

**DOI:** 10.3390/pharmaceutics14030680

**Published:** 2022-03-20

**Authors:** Deepal Vora, Amruta Dandekar, Sonalika Bhattaccharjee, Onkar N. Singh, Vivek Agrahari, M. Melissa Peet, Gustavo F. Doncel, Ajay K. Banga

**Affiliations:** 1 Center for Drug Delivery Research, Department of Pharmaceutical Sciences, College of Pharmacy, Mercer University, Atlanta, GA 30341, USA; deepal.vora@live.mercer.edu (D.V.); amrutaarun.dandekar@live.mercer.edu (A.D.); sonalika.arup.bhattaccharjee@live.mercer.edu (S.B.); 2 CONRAD, Eastern Virginia Medical School, Norfolk, VA 23507, USA; onsingh2@yahoo.com (O.N.S.); vagrahari@conrad.org (V.A.); mpeet@conrad.org (M.M.P.); doncelgf@evms.edu (G.F.D.)

**Keywords:** raloxifene, breast cancer, in vitro permeation testing, transdermal delivery, transdermal gel, chemical enhancer

## Abstract

Raloxifene (RLX) is a second-generation selective estrogen receptor modulator approved for the prevention of invasive breast cancer in women. Oral therapy of RLX requires daily intake and is associated with side effects that may lead to low adherence. We developed a weekly transdermal delivery system (TDS) for the sustained delivery of RLX to enhance the therapeutic effectiveness, increase adherence, and reduce side effects. We evaluated the weekly transdermal administration of RLX using passive permeation, chemical enhancers, physical enhancement techniques, and matrix- and reservoir-type systems, including polymeric gels. In vitro permeation studies were conducted using vertical Franz diffusion cells across dermatomed human skin or human epidermis. Oleic acid was selected as a chemical enhancer based on yielding the highest drug delivery amongst the various enhancers screened and was incorporated in the formulation of TDSs and polymeric gels. Based on in vitro results, both Eudragit- and colloidal silicon dioxide-based transdermal gels of RLX exceeded the target flux of 24 μg/cm^2^/day for 7 days. An infinite dose of these gels delivered 326.23 ± 107.58 µg/ cm^2^ and 498.81 ± 14.26 µg/ cm^2^ of RLX in 7 days, respectively, successfully exceeding the required target flux. These in vitro results confirm the potential of reservoir-based polymeric gels as a TDS for the weekly administration of RLX.

## 1. Introduction

In 2021, an estimated ~282,000 new cases of invasive breast cancer (BC) were expected to be diagnosed in women [1]. For women in the US, BC death rates are higher than those of any other cancer, besides lung cancer, and about 43,600 women were expected to die in 2021 from the disease [1]. Aberrant cell growth leading to a malignant tumor in breast cells leads to BC. Damage to DNA or mutations in specific genes (e.g., BRCA1, BRCA2, HER2, PALB2, PIK3CA) are believed to be the primary causes of BC, which is also commonly linked to estrogen exposure [2]. Local treatment options such as surgery or radiation or the use of systemic treatments including chemotherapy, hormone treatment, or targeted drug therapy are the main approaches used to treat BC. Besides the treatment options, various chemoprevention strategies are available to reduce the risk of BC [3]. Historically, BC prevention strategies were focused on patient awareness and screening, but given the rise in BC cases and incidence rates, a focus on targeted biomedical prevention has become more widespread and a preferred strategy in certain populations [4]. For high-risk individuals, prevention is limited to surgical approaches (i.e., bilateral mastectomy and salpingo-oophorectomy) or chemoprevention strategies using selective estrogen receptor modulators (SERMs) or aromatase inhibitors [5]. However, only 10% of women eligible for chemopreventive (daily oral) medications use them [6], partly because prevention options are burdensome and have undesirable side effects [7]. 

Tamoxifen (TMX), an FDA-approved medication for this indication, is safe and effective with a risk reduction of approximately 50% in those at high risk [8]. However, uptake and adherence to daily oral TMX are relatively low [5]. Raloxifene (RLX), another SERM like TMX, was originally developed to treat osteoporosis in postmenopausal women [5], but in 2007, the US Food and Drug Administration (FDA) approved the use of RLX to reduce invasive BC risk in postmenopausal women with osteoporosis and/or at high risk for BC [9,10]. Compared to TMX, RLX has a preferable safety profile with no increased risk for endometrial cancer and less risk for thromboembolism [5], making it an ideal first-line drug to use in a sustained delivery platform for chemoprophylaxis. Additionally, unlike TMX, RLX does not need to be converted into active metabolites to display potent activity [11].

RLX is commonly administered as a 60 mg daily oral tablet (EVISTA^®^), but owing to its low aqueous solubility and systemic absorption, its absolute bioavailability is only around 2% [12]. RLX predominantly undergoes fecal excretion, and its average plasma elimination half-life ranges from 27 to 32 h. Steady-state plasma concentrations associated with efficacy show medians ranging from 1.2 to 2.5 ng/mL [12]. Considering these targets, we hypothesized that effective plasma concentrations of RLX may be achieved and maintained for a longer duration (minimum of 1 week) through the enhanced systemic administration provided by a transdermal delivery system (TDS). Transdermal delivery of chemopreventive drugs can offer many advantages, including bypassing the first-pass metabolism and sustained delivery over an extended period, in addition to effective chemoprevention [13]. The transdermal route allows for drug substances to reach the systemic circulation directly across the skin barrier, thereby increasing bioavailability, and eliminating peak and trough plasma concentrations, which are usually associated with oral and injectable drug delivery. It further avoids the degradation of labile drugs in the gastrointestinal tract and, in general, is non-invasive and more patient-compliant [13].

RLX is a lipophilic molecule, having a log partition coefficient (LogP) of 5.5 [14], which makes its delivery across the skin challenging. Several formulation strategies [15,16,17,18,19,20] have been explored previously for transdermal administration of RLX. These include the development of various vesicular delivery systems such as nano-transferosomes [15,20] made of phospholipids and ethosomes [17] for enhanced transdermal delivery of RLX. In addition, a nanoparticle-based delivery system [16] and microneedle-based [21] physical enhancement technique [21] have also been investigated. However, the development of a once-weekly formulation of RLX has not been successfully demonstrated to date. Based on RLX’s absolute bioavailability of ~2% from a 60 mg daily oral tablet, as well as its half-life and clearance, we predict an estimated ~1.2 mg/day delivery of RLX needed transdermally to achieve targeted plasma concentrations [13,22]. For a daily transdermal dose of 1.2 mg, assuming a 50-cm^2^ patch size, the required target delivery calculated for 7 days is 168 µg/cm^2^. Our study aimed to evaluate a weekly transdermal delivery of RLX via pressure-sensitive adhesives (PSAs) and polymeric gels, ultimately to be used for chemoprophylaxis of BC. The overall goal of this product development process is to enhance RLX bioavailability, therapeutic effectiveness, adherence, and local tissue activity while reducing side effects, in comparison to standard oral delivery. Additionally, by providing weekly, sustained, systemic, and potentially localized delivery to breast tissue, better adherence and effective chemoprevention of BC may be achieved with a lower incidence of systemic side effects [23,24].

## 2. Materials and Methods

### 2.1. Materials

Raloxifene (free base and hydrochloride salt form) was purchased from Cayman Chemicals (Ann Arbor, MI, USA). Oleic acid and Transcutol^®^P were received from Croda Inc. (Edison, NJ, USA) and Gattefoss’e (Paramus, NJ, USA), respectively. Acetonitrile and methanol (Pharmco-Aaper, Brookfield, CT, USA); VOLPO^TM^ (Brij^®^O20: Polyoxyethylene (20) oleyl ether) and trifluoroacetic acid (Sigma Aldrich, St Louis, MO, USA); Eudragit^®^S 100 (Methacrylic acid-methyl methacrylate copolymer (1:2)) and colloidal silicon dioxide (CSD) (Evonik Industries, Wesseling, Germany); Dimethyl sulfoxide (DMSO) (Gaylord Chemical company, L.L.C, Tuscaloosa, AL, USA); and propylene glycol (PG) (Ekichem, Joliet, IL, USA) were procured. Acrylate PSA (DURO-TAK 87-2516) and polyisobutylene (PIB) PSA (DURO-TAK 87-6908) were received from Henkel Corporation (Dusseldorf, Germany); silicone PSA (BIO-PSA 7-4301) was from Dow Corning Corporation (Washington, DC, USA). The release liner (3M Scotchpak™ 1022 Fluoropolymer Coated Polyester Film) and backing membrane (3M™ CoTran™ Ethylene Vinyl Acetate Membrane Film 9707) were from 3M (St. Paul, MN, USA); Loparex Silicone coated PET film 7300A was from Loparex (Cary, NC, USA). Dermatomed human skin was procured from a skin bank (New York, NY, USA).

### 2.2. Methods

#### 2.2.1. Preliminary Studies

Preliminary studies using the salt form of RLX (RLX-HCl) were conducted to evaluate the passive permeation, pH-dependent diffusion, and microneedle-mediated and iontophoretic delivery of RLX-HCl. In vitro permeation testing (IVPT) was conducted using dermatomed human or dermatomed porcine ear skin where polyethylene glycol (PEG) 400:PBS::4:6 was used as a receptor solution based on the solubility studies of RLX-HCl and to maintain the sink conditions. All studies were conducted using a 90% saturated solution of RLX-HCl in respective vehicles. Passive permeation was tested using PG as a vehicle, and pH-dependent diffusion was checked at pH 3.5 and 8.5 using a 1:1 (%*v/v*) solution of PG and citrate (pH 3.5) or borate (pH 8.5) buffer. Microneedle-assisted delivery was tested using skin pre-treated with maltose microneedles for 2 min, whereas anodal iontophoresis was conducted for 4 h at 0.5 mA/cm^2^ using a published protocol [25] with 1:1::PG: citrate buffer. All studies were conducted for 7 days except for iontophoresis, which was conducted for 24 h.

#### 2.2.2. Solubility and Stability Studies

The solubility of RLX was determined in 10 mM PBS, 4:6:: PEG 400: PBS, 6% *w*/*v* Volpo^TM^ in citrate buffer (10 mM, pH 3), and 50% ethanol in citrate buffer (10 mM, pH 3) to select the receptor solution. Additionally, the solubility was also screened in the presence of various chemical enhancers, including PG, oleic acid, DMSO, and Transcutol^®^P. To determine the solubility, an excess amount of drug was added to 500 µL of each vehicle followed by overnight shaking at room temperature. After that, the solution was centrifuged at 13,400 rpm for 15 min. The supernatant was filtered, diluted, and analyzed by ultra-performance liquid chromatography (UPLC) to determine the solubility. In addition to its solubility, the stability of RLX in the above receptor solutions and in PG and DMSO was also studied to analyze for any degradation. For this, a specified amount of RLX was dissolved in each vehicle. The samples were incubated at 37 °C and analyzed by the UPLC method at 0, 24, 72, 120, and 168 h time points to determine the amount of RLX.

#### 2.2.3. Skin Preparation for IVPT Studies

Heat-separated human epidermis and dermatomed human cadaver skin were used for IVPT studies. Human epidermis was freshly isolated by first immersing dermatomed skin in 10 mM PBS at 60 °C for 2 min, separated using forceps and a spatula, and then cut into suitable pieces to be mounted on the Franz diffusion cells. For IVPT studies using dermatomed human skin, the skin was thawed in 10 mM PBS at 37 °C and cut into pieces. 

#### 2.2.4. Skin Integrity Testing

Skin barrier integrity was assessed by measuring electrical resistance using a published method [25]. A silver/silver chloride electrode connected to the waveform generator and digital multimeter (Agilent Technologies, Santa Clara, CA, USA) was used. Pieces of human epidermis or dermatomed skin were mounted on a Franz cell containing 5 mL of 10 mM PBS in the receptor compartment. The skin tissue was equilibrated for 15 min after adding 300 µL of 10 mM PBS to the donor chamber. The voltage drop across the skin was then measured by dipping silver wire and silver chloride in the receptor and donor compartments, respectively. The resistance was calculated in kΩ using the following equation [25]:

$$R_{S}=V_{S}R_{L\;}/\left(V_{O}-V_{S}\right)$$

The load resistor (*R_L_*) and *V_O_* were 100 kΩ and 100 mV, respectively. Skin samples having a resistance >10 kΩ were used for the permeation study. 

#### 2.2.5. In Vitro Permeation Testing (IVPT)

IVPT on vertical static Franz diffusion cells was performed to determine the rate and extent of delivery of RLX into and across porcine ear skin (dermatomed) and human skin (dermatomed skin and heat-separated epidermis). The dermatomed skin was thawed and cut to size, and the thickness was recorded. The skin was then mounted between the donor and receptor compartments of the Franz diffusion cells with the stratum corneum side facing up. The receptor compartment contained 5 mL of receptor solution (6% *w*/*v* VOLPO™ in citrate buffer of pH 3). After dosing, receptor samples were taken at predetermined time points (0, 2, 4, 8, 24, 48, 72, 96, 120, 144, and 168 h) and analyzed for the amount of RLX using the UPLC method. An equal amount of receptor solution was added at each time point to maintain the sink condition. At the end of the IVPT study with dermatomed skin, the permeation area was cut out, and skin was minced into three pieces, followed by the addition of methanol to extract RLX from the skin, and quantified using UPLC. Passive diffusion of RLX across human epidermis for 7 days was evaluated from a 9 mg/mL solution of RLX in PG (300 µL).

#### 2.2.6. Screening of Chemical Enhancers

The effect of chemical enhancers on the delivery of RLX was tested across dermatomed human skin using 100 µL of 90% saturated solution of RLX in combinations with different chemical enhancers, including 10% oleic acid in PG, 5% oleic acid + 40% Transcutol^®^P in PG, 5% oleic acid + 10% Transcutol^®^P + 15% DMSO in PG, and 45.50% DMSO in PG (all in % *w/w*).

#### 2.2.7. Drug-in-Adhesive PSA-Based TDS

To develop a solution-based PSA TDS, the solubility of RLX in blends of different PSAs (acrylate, silicone, and PIB) with 10% *w/w* oleic acid was determined using slide crystallization studies. However, due to the very low solubility of RLX in PSA–oleic acid blends (<0.10% *w/w*), the required drug loading was not achieved using a solution-based TDS; hence, a suspension-based TDS was developed. For the suspension-based PSA TDS, three methods (slow rotary mixing, bead mill homogenization, and probe homogenization) were tested to produce a uniform suspension. Heptane was used as a processing solvent to make up the bulk volume required for probe homogenization. PIB PSA was excluded due to difficulty in homogenizing the high-viscosity adhesive blend.

The optimized method for formulating the suspension-based PSA TDS included probe homogenizing RLX with PSA and heptane at 32,000 rpm for 5 min. Further, a weighed amount of oleic acid was added to the slurry and probe homogenized at 32,000 rpm for 2 min. This uniform suspension of RLX and oleic acid was then blended with PSA at 15,000 rpm for 2 min, followed by slow rotary shaking for 15 min to remove the air bubbles. The blend was then cast on the release liner and allowed to air dry for 45 min. Suitable backing membranes and release liners were selected for acrylate and silicone suspension-based PSA TDSs. For the selection of release liners, the blend was cast on different release liners and allowed to air dry. A gloved hand was then used to test the peeling and adhesiveness of the formulations on individual membranes. The coat weight and drug content for both PSA TDSs were analyzed. The TDSs were punched to a size large enough to cover the permeation area on Franz diffusion cells. The IVPT study using these punched TDSs [24] was conducted for 7 days to determine the amount of RLX delivered across heat-separated human epidermis.

#### 2.2.8. Polymeric TDS

Various polymers such as polyvinylpyrrolidone, polyvinyl alcohol, Carbopol, and Eudragit were screened for the formulation of a matrix-type TDS. As oleic acid was required for enhanced delivery of RLX, a polymer (Eudragit^®^S100) soluble in the oleic acid and PG blend was selected for formulating the TDS. RLX (3.50% *w/w*) was dissolved in a blend of 8.50% *w/w* oleic acid and 73% *w/w* PG. Eudragit^®^S100 (15% *w/w*) was then added to the blend and allowed to dissolve overnight on a slow rotary mixer (Preiser Scientific Inc., St. Albans, WV, USA). Ethanol (600 µL for a 5 g blend) was added as a processing solvent for casting the TDS. The homogenous blend was then cast on the backing membrane using a casting knife, dried to allow the evaporation of ethanol, and laminated with the release liner. IVPT was conducted to determine the amount of RLX delivered across heat-separated human epidermis.

#### 2.2.9. Polymeric Transdermal Gels

The polymeric transdermal gels were prepared using two polymers, Eudragit^®^S100 and colloidal silicon dioxide (CSD). Oleic acid was incorporated to enhance the delivery of RLX. To prepare gels, RLX was first dissolved in a mixture of oleic acid and PG using a tissue homogenizer (THq, Omni International, Kennesaw, GA, USA). The required quantity of each polymer was then added to the solution and blended overnight on a low-shear rotating mixer (Preiser Scientific Inc., St. Albans, WV, USA) to obtain a clear gel. Table 1 shows the composition of the formulated polymeric gels.

The polymeric gels were characterized for their pH and rheology [26]. The pH of gels was determined using a pH meter for semi-solids (SympHony B30PCI pH meter; VWR, Radnor, PA, USA). Rheological evaluation of the prepared polymeric transdermal gels was conducted using a parallel-plate rheometer (Modular Compact Rheometer, MCR 302, Anton Paar Germany GmbH, Ostfildern, Germany). A flow curve was plotted and an amplitude sweep test was performed for both gels. The flow curve was plotted to determine how the gel behaves when subjected to changing shear rates, while the amplitude sweep used increasing amplitude to probe gel properties such as rheological stability. Rheoplus/32 V3.62 software was used to analyze the data. 

Delivery of RLX from both finite and infinite doses of polymeric gels was evaluated. Approximately 10 mg/cm^2^ of semi-solid formulation is considered a finite dose [27]. Thus, for a finite dose, 10 µL of gel was applied on the permeation area, whereas 200 µL was used for infinite dosing. Permeation of RLX from these gels across heat-separated human epidermis was tested using the same protocol as mentioned for passive permeation. 

#### 2.2.10. Quantitative Analysis of RLX

A reverse-phase UPLC method was developed and validated for quantitative analysis of RLX. A Waters Acquity H-Class UPLC coupled with a photodiode array detector was used. An Acquity UPLC BEH C18 1.7 micron, 2.1 × 50 mm column was used. An isocratic elution with a flow rate of 0.9 mL/min at a solvent composition of 30: 70:: acetonitrile +0.10% TFA: DI water +0.10% TFA and a run time of 3 min was used. The injection volume was 0.5 µL, and the detection wavelength was 287 nm. The retention time for RLX was found to be at 0.6 min. The developed UPLC method was validated for linearity (R^2^ = 0.99999), and interday and intraday accuracy and precision. The limit of detection was 0.03 µg/mL, while the limit of quantification was 0.08 µg/mL.

#### 2.2.11. Data Analysis

All studies were conducted in replicates of 4 or more. Results were calculated as the mean value ± standard error (SE). Statistical calculations were performed using GraphPad Prism version 8.03. Statistical differences were determined using one-way analysis of variance (ANOVA) and a Kruskal–Wallis test. In addition, post hoc tests were carried out to calculate the statistical difference between groups. A *p*-value of less than 0.05 (*p* < 0.05) was considered statistically different.

## 3. Results

### 3.1. Preliminary Studies

There was no passive permeation of RLX-HCl observed from PG in 7 days. Further, no passive delivery was observed with an alteration in the pH (pH 3.5 and 8.5). Thus, the pH did not affect the transdermal delivery of RLX-HCl. Both pre-treatment with maltose microneedles to breach the epidermis and anodal iontophoresis resulted in increased delivery of RLX-HCl, with a total delivery of 13.56 ± 3.78 µg/cm^2^ and 45.87 ± 9.25 µg/cm^2^, respectively.

### 3.2. Solubility and Stability Studies

**Figure 1:**
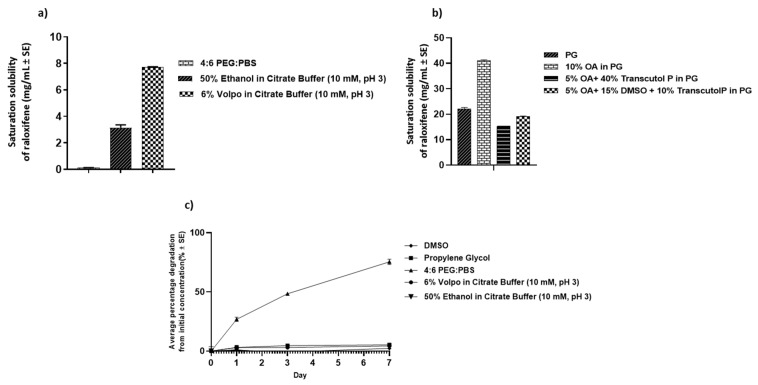
Solubility and stability studies: (**a**) solubility of RLX in receptor solutions; (**b**) solubility of RLX in combination with chemical enhancers; (**c**) stability profile of RLX in various vehicles at 37 °C; *n* = 4; PEG: polyethylene glycol 400, PBS: phosphate-buffered saline, PG: propylene glycol, OA: oleic acid, DMSO: dimethyl sulfoxide.

Solubility and stability studies were conducted to select a suitable receptor solution for IVPT studies. The solubility of RLX in 10 mM PBS was <1 mg/mL, which was not enough to maintain sink conditions. Among the other receptor solutions screened (Figure 1a), 6% *w*/*v* VOLPO in citrate buffer showed the highest solubility of 7.71 ± 0.70 mg/mL, which was enough to maintain sink conditions. The literature also reports the use of such solutions for lipophilic compounds [28]. Stability studies indicated significant degradation of RLX in the vehicle containing 4:6::PEG 400: PBS, but RLX was found to be stable in other vehicles, including 6% *w*/*v* VOLPO in citrate buffer (pH 3). Thus, based on the solubility and stability data, 6% *w*/*v* VOLPO in citrate buffer was selected as the receptor solution for permeation studies. Solubility studies showed increased solubility of RLX with the incorporation of chemical enhancers, and 10% oleic acid in PG showed the highest solubility of 41.08 ± 0.31 mg/mL (Figure 1b). Stability studies showed no degradation of RLX in PG and DMSO (Figure 1c).

### 3.3. Passive Permeation

Passive delivery of RLX from PG at the end of 7 days was only 0.18 ± 0.18 µg/cm^2^. The low permeability could be due to the lipophilic nature of RLX and the molecular weight falling on the higher side of the ideal range of <500 Da for transdermal delivery. 

### 3.4. Screening Chemical Enhancers

**Figure 2:**
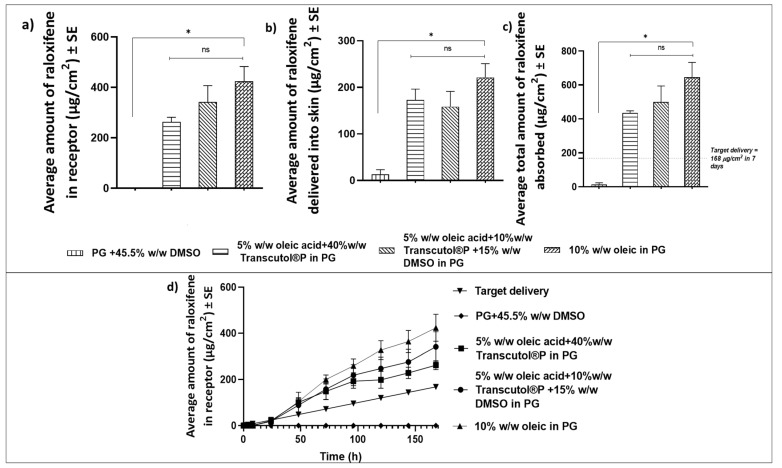
Screening of chemical enhancers for delivery of raloxifene: (**a**) cumulative amount of raloxifene delivered into the receptor after 7 days; (**b**) amount of raloxifene delivered into the skin at the end of 7 days; (**c**) total amount of raloxifene delivered into and across the skin in 7 days; (**d**) permeation profile of raloxifene. Statistical analysis was performed with a Kruskal–Wallis test. ns indicates no significant difference between groups (*p* > 0.05); * indicates a significant difference between groups (*n* = 4, *p* < 0.05).

The total delivery of RLX from (all in weight%) 10% oleic acid in PG, 5% oleic acid + 40% Transcutol^®^P in PG, and 5% oleic acid + 10% Transcutol^®^P + 15% DMSO in PG was 645.01 ± 88.07 µg/cm^2^, 435.18 ± 12.65 µg/cm^2^, and 500.18 ± 94.30 µg/cm^2^, respectively (Figure 2). The other three combinations, except for 45.50% DMSO in PG, exceeded the target delivery required for 7 days. The total amount of RLX delivered into and across the skin (645.01 ± 88.07 µg/cm^2^) from a donor solution of 10% oleic acid in PG was the highest and thus was selected for further formulation development of a matrix-type PSA-based TDS.

### 3.5. PSA-Based TDS

**Figure 3:**
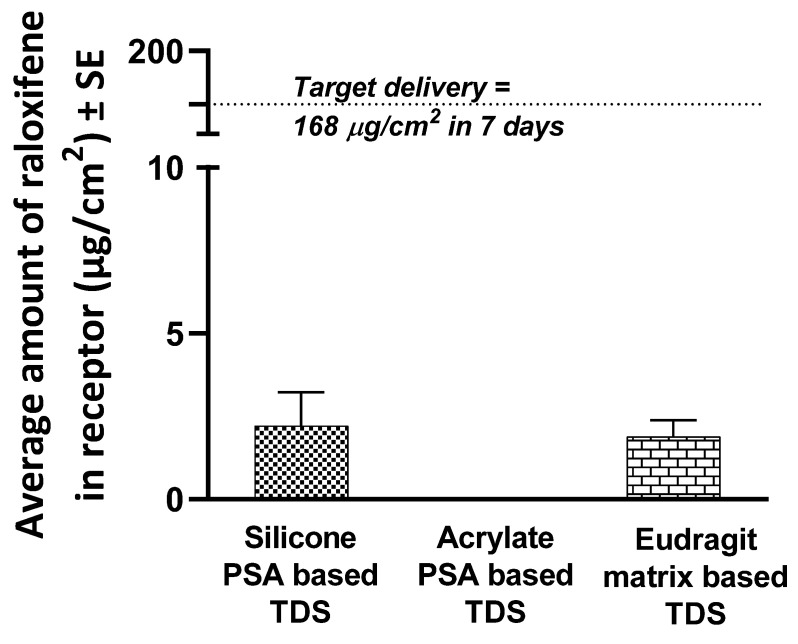
Total amount of raloxifene delivered into the receptor from PSA-based TDS and polymeric Eudragit-based TDS, *n* = 4.

For the formulation development of a matrix-type PSA-based TDS, among the three methods tested (slow rotary mixing, bead mill homogenization, and probe homogenization), probe homogenization was selected to give a uniform suspension of RLX in PSAs. A 3M Scotchpak™ 1022 Release Liner Fluoropolymer Coated Polyester Film was used as a release liner based on the observation that it provided the least residue on removal. Based on the adhesiveness of the blend to backing membranes, 3M™ CoTran™ Ethylene Vinyl Acetate Membrane Film 9707 was selected as the backing membrane for the acrylate PSA suspension-based TDS, whereas Loparex Silicone coated PET film 7300A was selected as the backing membrane for the silicone PSA suspension-based TDS. The thickness of 30 mil for the acrylate TDS and 25 mil for the silicone TDS was optimized to give a target coat weight of 200 GSM (gram per square meter) for both adhesive systems. The amount of RLX permeated from the TDSs with silicone (2.21 ± 1.02 µg/cm^2^) and acrylate (0.0 ± 0.0 µg/cm^2^) PSAs did not meet the desired target delivery (Figure 3). 

### 3.6. Polymeric TDS

A 3M™ CoTran™ 9720 Silicone coated PET film was selected as the backing membrane, while 3M Scotch Pak™ 1022 Release Liner Fluoropolymer Coated Polyester Film was selected as a release liner for the polymeric TDS. As shown in Figure 3, the amount of RLX delivered into the receptor from the Eudragit^®^ S100 polymeric TDS in 7 days was 1.89 ± 0.50 µg/cm^2^, significantly lower than the target delivery required. 

### 3.7. Polymeric Gels

**Figure 4:**
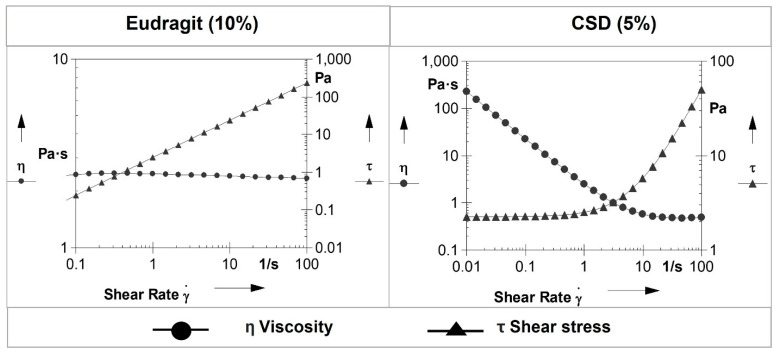
Rheological characterizations of gels: flow curves of Eudragit (10%) and CSD (5%) gels.

**Figure 5:**
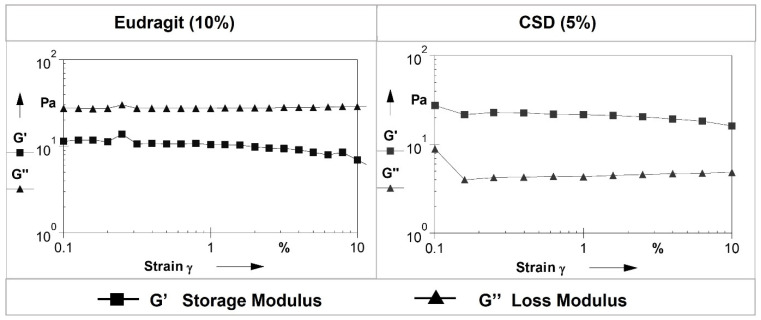
Rheological characterizations of gels: amplitude sweep tests of Eudragit (10%) and CSD (5%) gels.

The pH of Eudragit (10%) and CSD (5%) gels was 5.85 ± 0.01 and 6.15 ± 0.01, respectively. The results of the rheological evaluations of both gels are presented in Figure 4 and Figure 5. The storage modulus measures the stored energy representing the elastic response of a material, whereas the loss modulus measures the heat dissipated by the material representing the viscous response of a material [29]. The flow curves for the Eudragit (10%) and CSD (5%) gels (Figure 4) showed that with increasing shear rate, the viscosity of the Eudragit and CSD gels was decreased. Figure 5 indicates the rheograms from the amplitude sweep test, and the Eudragit (10%) gel had a higher loss modulus as compared to the storage modulus, indicating a flowy gel, whereas the CSD (5%) gel showed a higher storage modulus than the loss modulus, indicating a less flowy gel. 

**Figure 6:**
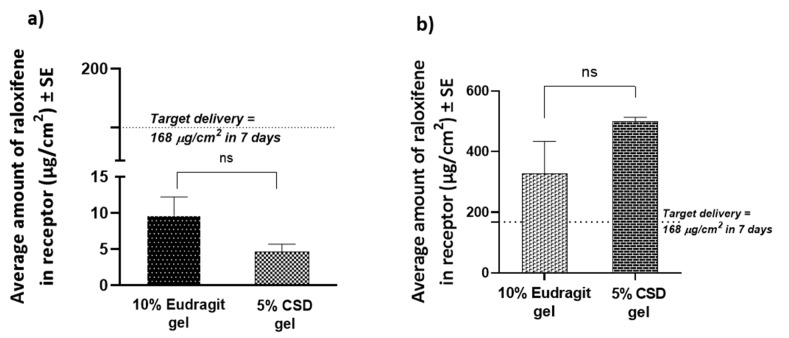
Cumulative amount of raloxifene delivered into the receptor in 7 days from (**a**) finite dosing of gels and (**b**) infinite dosing of gels. Statistical analysis was performed with a Mann–Whitney test. ns indicates no significant difference between groups (*n* = 4, *p* > 0.05).

**Figure 7:**
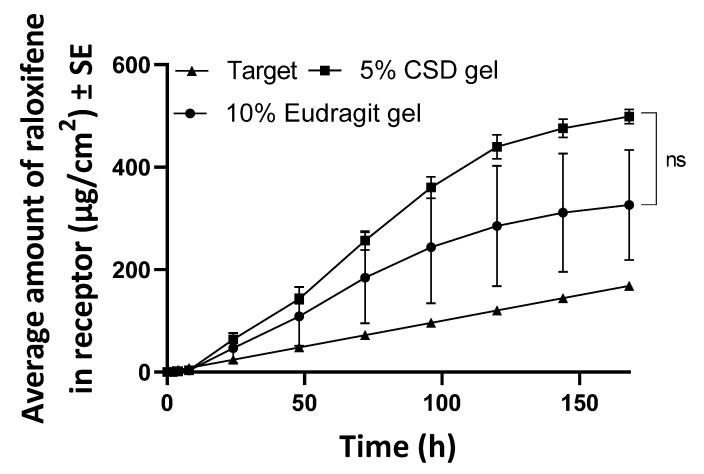
Permeation profile of raloxifene from infinite dosing of gels. Statistical analysis was performed with a Mann–Whitney test. ns indicates a significant difference between groups (*n* = 4, *p* > 0.05).

The observed total delivery of RLX in 7 days from a finite dose of the Eudragit^®^ S100 and CSD gels was 9.63 ± 2.58 µg/cm^2^ and 4.56 ± 1.13 µg/cm^2^, respectively, (Figure 6). Thus, a finite dose of the gels did not deliver the required amount of RLX; however, an infinite dose of the Eudragit^®^ S100 and CSD gels delivered a total of 326.23 ± 107.58 µg/cm^2^ and 498.81 ± 14.26 µg/cm^2^ in 7 days, respectively, achieving the desired target delivery. The permeation profile of RLX from infinite gel dosing showed a continuous increase, as shown in Figure 7.

## 4. Discussion

This research project focused on developing transdermal formulations of RLX, an osteoporosis and BC prevention agent for women. The selection of an appropriate receptor solution is essential for determining the in vitro delivery of highly lipophilic compounds such as RLX. The commonly used receptor solutions such as saline may underestimate results due to the low water solubility of the drug and the inability of the solution to appropriately maintain sink conditions. The use of ethanol, PEG 400, and VOLPO as receptor solutions has been described in the literature for lipophilic drugs [28]. Therefore, we tested RLX solubility and stability in the above receptor solutions. PEG 400:PBS was selected as a receptor solution in preliminary studies based on its sufficient solubility to maintain sink conditions [28]. However, receptor solutions containing PEG 400 showed significant degradation (~50%) in 7 days, and the solubility of RLX in 50% ethanol in PBS was not enough to maintain sink conditions. The potential reason for the observed degradation of RLX in the PEG 400:PBS receptor solution could be due to the presence of hydroperoxide in PEG 400, leading to oxidative degradation of RLX as seen in similar studies of RLX with excipients containing hydroperoxide [30,31]. VOLPO (6% *w*/*v*) was selected to be the receptor solution in our studies since it maintained sink conditions for RLX while keeping it stable in this solution for 7 days.

Preliminary studies performed with RLX-HCl showed promising results using physical enhancement techniques. These physical permeation enhancement techniques are powerful but complex, and their practicability for transdermal application of BC chemoprevention agents is low. Hence, other formulation approaches such as TDSs and polymeric gels were explored for better patient compliance and ease of application. Nagai et al. successfully designed a transdermal formulation containing RLX nanoparticles for osteoporosis treatment. They observed that the incorporation of menthol as a penetration enhancer significantly enhanced the delivery of RLX nanoparticles through the skin [16]. In our studies, the effect of chemical enhancers on the delivery of RLX was tested by varying concentrations of oleic acid, DMSO, and Transcutol^®^P. Comparing these different combinations, the degree of saturation of RLX in each combination was maintained at the same saturation (90% saturation) to maximize the thermodynamic activity in all. This ensured the maximum enhancement effect from each vehicle. Each of these enhancers can act differently in different formulations with varying vehicles and can exhibit an effect via different mechanisms reported extensively in the literature [32]. Oleic acid and DMSO both work as chemical enhancers by altering the structure of the skin’s layers, thereby acting on its barrier function [33]. Transcutol^®^P acts as a drug solubilizer and can easily penetrate the stratum corneum and interact with the intracellular fluid to modify stratum corneum permeability [34], whereas PG can, itself, act as an enhancer. We studied possible synergistic effects of PG in combination with oleic acid, Transcutol^®^P, and DMSO. The literature reports synergistic effects of oleic acid along with Transcutol^®^P in some studies [35]. In our studies, the addition of Transcutol^®^P (both 10 and 40%) to oleic acid resulted in increased total delivery of RLX, although the difference was not statistically significant (Figure 2). In contrast, a higher concentration of oleic acid (10%) alone in PG significantly enhanced the delivery of RLX, with the highest total delivery. This can be attributed to the greater solubility of RLX in 10% oleic acid in PG as compared to other vehicle combinations (Figure 1). In our study, delivery of RLX was successfully enhanced for formulations containing oleic acid and Transcutol^®^P, which met the target delivery required for 7 days. However, the group containing 45.50% DMSO in PG did not meet the target delivery, which contrasts with several other studies that showed enhanced drug delivery using DMSO [32]. Possible reasons for lower delivery could be the higher affinity of RLX in DMSO, which did not allow the drug to permeate across the skin. Based on the results of our screening, oleic acid was selected as the chemical enhancer to be incorporated in TDSs for RLX.

**Figure 8:**
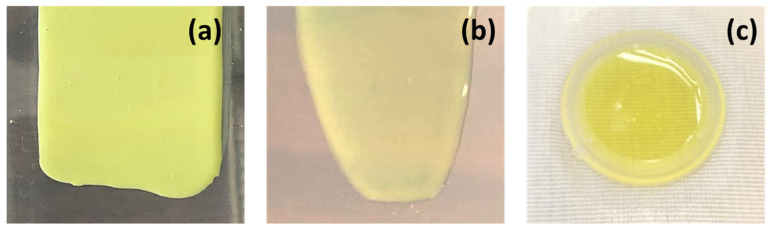
Images of raloxifene loaded in (**a**) a suspension-based PSA TDS, (**b**) a polymer-based TDS, and (**c**) polymeric transdermal gels.

We first formulated a suspension-based drug-in-adhesive matrix-type TDS for weekly administration of RLX and tested it for IVPT (Figure 8). However, the amount of RLX permeated from the matrix-type TDSs with silicone and acrylate PSAs did not meet the desired target delivery. Since the PSA-based TDSs could not deliver the required amount of RLX, other polymers were screened to formulate a matrix-type polymeric TDS. Eudragits are polymethacrylate-based copolymers used to formulate matrix-type TDSs because of their film-forming and adhesive nature [36]. Chantasart et al. formulated and evaluated Eudragit^®^ polymeric films for transdermal delivery of piroxicam. They demonstrated the use of Eudragit^®^ for the formulation of TDSs [36]. We formulated an RLX polymeric TDS using the Eudragit^®^S100 polymer (Figure 8b). However, like the suspension-based PSA TDS, the delivery of RLX from the polymeric TDS was also very low. These results indicate that a matrix-type TDS might not be suitable for the weekly administration of RLX. Hence, we then explored reservoir-type polymeric transdermal gels of RLX (Figure 8c). The pH of the formulation applied on the skin is an essential factor to be considered since it may affect the irritation properties of the formulation and also impact the dermal absorption of the actives. [28]. The pH of the skin is widely known to be acidic (~5.5) for the maintenance of the stratum corneum and barrier permeability [37]. The pH of our formulated gels was also on the acidic side and within the acceptable range (pH 4–6) for formulations applied on the skin [37]. 

Further characterizations were conducted to understand the rheology of the formulated gels. Both gels presented shear-thinning properties, which is ideal for topical gel application [38]. The higher loss modulus of the Eudragit gel indicated a less viscous gel, whereas the higher storage modulus than the loss modulus for the CSD gels indicated the gels’ ability to store deformation in an elastic manner. Both finite and infinite doses of the formulated polymeric gels were tested by IVPT, and the infinite dose of gels successfully achieved the target delivery required for 7 days. The higher delivery observed from an infinite dose than a finite dose of gel can be due to various factors. For example, evaporation of formulation excipients leading to metamorphosis and drying of the formulation on the skin was observed in the case of the finite dose, which could be one of the reasons for the low delivery of RLX. Thus, applying these gels as a typical semi-solid formulation (finite dose) may not be a practical strategy to achieve the desired therapeutic concentration. On the other hand, the infinite dose showed significantly higher delivery, which can be due to the occlusive effect observed in the case of the large volumes applied [39]. Infinite dosing is generally recommended for pharmacokinetics evaluation, whereas finite dosing is important to determine drug distribution in the skin [39]. This study indicates that an infinite dose of these gels can be used for sustained systemic delivery of RLX over 7 days. Thus, these results from in vitro studies confirm the feasibility of the weekly administration of RLX using polymeric gels in a higher volume in a reservoir-based TDS for the prevention of breast cancer. 

## 5. Conclusions

This study aimed to develop a weekly transdermal formulation for sustained delivery of RLX to ultimately enhance its adherence and therapeutic effectiveness while reducing side effects. Various formulation strategies, including passive permeation, use of chemical enhancers, physical enhancement techniques, PSA- and polymer-based matrix-type systems, and polymeric transdermal gels, were tested. Among the strategies explored, Eudragit- and CSD-based transdermal gel formulations of RLX exceeded the target skin flux, achieving adequate transdermal delivery for at least 7 days. These in vitro results confirm the potential for transdermal gels to be used as a reservoir-based TDS approach for the weekly administration of RLX.

## Figures and Tables

**Table 1:** Composition of polymeric transdermal gels for raloxifene.

Contents	Eudragit^®^S100 Gel	Colloidal Silicon Dioxide Gel
	Amount in % *w/w*	
Raloxifene	3.69%	3.90%
Propylene glycol	77.68%	82%
Oleic acid	8.63%	9.10%
Polymer	10%	5%

## Data Availability

Data presented in this study are contained in the manuscript.
